# Intraoperative Event Mechanisms During the Early Adoption of Yamane Flanged Intrascleral Intraocular Lens (IOL) Fixation: Insights From Surgical Video Review

**DOI:** 10.7759/cureus.115152

**Published:** 2026-08-25

**Authors:** Sindy B Sembiring, Stefenie Stefenie, Jessica Zarwan

**Affiliations:** 1 Department of Ophthalmology and Vision Sciences, Universitas Prima Indonesia, Medan, IDN; 2 Department of Vitreoretina, Sabang Merauke Eye Center, Medan, IDN; 3 Department of Ophthalmology, Faculty of Medicine Universitas Indonesia, Dr. Cipto Mangunkusumo Hospital, Jakarta, IDN

**Keywords:** aphakia, complications of secondary iol, crystalline lens dislocation, iol dislocation, secondary iol procedures, sutureless intrascleral fixation

## Abstract

Introduction

To identify intraoperative events during early Yamane adoption using structured surgical video review, linking each event to procedural stage, plausible mechanism, and recovery action.

Methods

This retrospective study included 24 eyes undergoing Yamane flanged intrascleral intraocular lens (IOL) fixation between November 2024 and February 2025, representing early adoption by a single vitreoretinal surgeon. All procedures were video-recorded and reviewed postoperatively by a non-operating co-author experienced in Yamane fixation using a structured micro-step checklist to time-stamp events, assign plausible mechanisms, and record recovery actions. Secondary outcomes included operative timing and postoperative safety and visual outcomes.

Results

Intraoperative events occurred in eight eyes (33.3%): haptic slippage/disengagement (five), haptic breakage (one), mild vitreous hemorrhage (one), and suprachoroidal hemorrhage (one). Slippage most commonly involved the leading haptic and clustered around the “let-go” phase (M7) and subsequent trailing-haptic manipulation, with recurrent contributors including inadequate threading length and optic torque during nondominant-hand docking. Most events were recovered intraoperatively through repositioning, re-threading, or scleral re-tunneling; IOL exchange was required in one case of haptic fracture. Mean corrected distance visual acuity improved from logarithm of the minimum angle of resolution (logMAR) 0.96 ± 0.79 preoperatively to 0.47 ± 0.55 postoperatively.

Conclusion

Structured video review with micro-step classification standardizes linking events to stage, mechanism, and recovery, providing a practical framework for mentors to deliver targeted feedback and recommend technical refinements during training. Although based on a single-surgeon early series, this work introduces the checklist and warrants validation in broader cohorts.

## Introduction

Visual rehabilitation in aphakia or inadequate capsular support remains challenging, with options ranging from retropupillary iris-claw intraocular lens (IOLs) to scleral-fixated IOLs, either sutured or sutureless [1,2]. The Yamane flanged intrascleral fixation places the IOL in a posterior chamber position through a minimally invasive, sutureless approach and has shown favorable outcomes [3]. However, the technique is technically demanding, and haptic-related intraoperative complications have been reported during early surgical experience with sutureless intrascleral IOL fixation [4]. Most outcome reports either omit the learning phase or lack analysis of where and why errors occur [5,6]. To optimize training and safety, early adopters benefit from objective methods for identifying error-prone steps, understanding their mechanisms, and guiding targeted refinements. Video-based review provides such an approach, enabling systematic assessment of intraoperative performance and evaluation of the surgical process beyond final visual outcomes [7,8].

This study seeks to identify and characterize intraoperative events and complication-prone steps during early adoption of the Yamane technique through systematic surgical video review using a structured checklist. The review focuses on when each event occurs, its plausible mechanism, and how it is managed intraoperatively. Surgical duration, postoperative complications, and visual acuity are evaluated as secondary outcomes.

## Materials and methods

Study design and ethics

This retrospective, single-center case series was conducted at the Sabang Merauke Eye Center (SMEC) in Medan, Indonesia. All Yamane procedures were performed by a single vitreoretinal surgeon (S.S.), who had no prior clinical experience performing the Yamane technique before the first case included in this series. This study was conducted in accordance with the Declaration of Helsinki and was approved by the SMEC Research Ethical Review Committee (Approval No. EA00000694).

Eligibility

Inclusion 

Consecutive eyes that underwent flanged intrascleral IOL fixation (Yamane technique) between November 2024 and February 2025, representing the surgeon's early adoption. Indications included aphakia without adequate capsular support, dislocated IOL, subluxated IOL, subluxated lens, dislocated lens, and posterior capsule rupture.

Exclusion

For the intraoperative analysis, cases lacking complete surgical video were excluded. For secondary outcomes analysis, eyes with <1-month follow-up were excluded from those specific analyses.

Baseline data 

From the medical records, baseline data included age, sex, laterality, surgical indication, systemic comorbidities, and preoperative ocular parameters. Biometry was performed using an optical biometer (NIDEK Co., Ltd., AL-Scan, Gamagori, Aichi, Japan). When reliable optical measurements could not be obtained because of a mature cataract, the optional built-in ultrasound biometer was used. IOL power was calculated using the Sanders-Retzlaff-Kraff/Theoretical (SRK/T) formula with a target refraction of −1.00 D. The biometry setting was selected according to the preoperative lens/IOL status: aphakic mode was used for aphakia and dislocated lens or IOL, phakic mode for subluxated lens, and pseudophakic mode for subluxated IOL.

Platform and intraocular lens (IOL)

All procedures were performed using a 27-gauge needle with a foldable three-piece IOL (PU6AS, Avansee, Kowa, Japan). Globe stabilization was maintained with a pars plana infusion cannula as part of a standard 23-gauge three-port pars plana vitrectomy system (Constellation Vision System, Alcon Laboratories, or Stellaris PC, Bausch & Lomb). Complete pars plana vitrectomy followed by prophylactic 360° barrage endolaser was performed in all eyes.

When indicated, a subluxated or dislocated lens was managed by phacoemulsification, transcorneal extraction, or endofragmentation, and subluxated or dislocated IOLs were removed transcorneally. The corneal incision configuration was adapted to the surgical scenario. The main corneal incision was generally positioned at approximately the 12 o'clock position, with a 2.8-mm incision typically used for insertion of the three-piece IOL. When explantation of a pre-existing IOL required a larger incision, the incision was enlarged to approximately 3.5 mm. Two corneal sideports were used, located at approximately the 2:30-o'clock and 10:30-o'clock positions. The three-piece IOL was then placed in the anterior chamber, with the trailing haptic kept outside to prevent posterior dislocation.

Angled sclerotomies were planned 2.0 mm posterior to the limbus and parallel to the iris plane. The first sclerotomy was positioned at approximately the 2:30-o'clock position, and the second was positioned 180° from the first, at approximately the 8:30-o'clock position. The entry sites were marked intraoperatively using a straight needle shaft as a linear reference to confirm opposing alignment. The intended intrascleral tunnel length was approximately 2 mm.

The 27-gauge needles were initially attached to syringes during haptic docking. The first angled sclerotomy was created at the marked entry site, and the leading haptic was docked into the lumen of the first needle. After leading-haptic docking, the first needle was detached from the syringe and temporarily placed on the conjunctiva during the “let-go” phase. The second angled sclerotomy was then created at the opposing marked entry site, and the trailing haptic was docked into the second needle lumen and externalized with the needle. The externalized trailing haptic was cauterized using low-temperature diathermy to create an approximately 0.3-mm flange, which was tucked into the scleral tunnel beneath the conjunctiva. The surgeon then returned to the leading haptic, externalized it, created a similar flange, and secured it within the opposite scleral tunnel to achieve symmetric IOL centration.

Haptic manipulation throughout the procedure was performed using a 23-gauge curved gripping forceps (FT 270106CU, Huaian Frimen Co., Ltd., Huai’an, Jiangsu, China) and a straight end-gripping forceps (Bausch + Lomb), with the choice of forceps adapted to the haptic position and intraoperative requirements. All corneal incisions were closed with 10-0 nylon sutures (FSSB Nylon Suture 10/0 Black, FSSB, Germany), and the pars plana sclerotomy ports were secured with 7-0 Vicryl (Ethicon, Somerville, NJ, USA). Peripheral iridectomy was not performed, and no tamponade was used.

Intraoperative video review 

All procedures were video-recorded and reviewed postoperatively using a structured checklist. The complete Structured Surgical Video Review Tool, including the micro-step map, event taxonomy, mechanism tags, corrective action categories, and event logging template, is provided in the Appendix (see Tables 3-7). Video assessment was performed by J.Z., who was experienced in Yamane fixation and was not the operating surgeon. For each case, intraoperative events were time-stamped and classified by micro-step (M1-M17; e.g., leading-haptic docking and threading, the “let-go” phase, trailing-haptic docking/threading, externalization, flanging, and intrascleral burial). For each event, J.Z. recorded the haptic involved (leading/trailing), presumed mechanistic contributors (e.g., forceps-haptic-needle axis misalignment, nondominant-hand ergonomics, inadequate threading length, optic rotation/torque, tunnel geometry, or bleeding with reduced visualization), and the immediate recovery action. Corrective maneuvers (e.g., re-threading and scleral re-tunneling) were recorded as recovery actions and were not classified as complications. An intraoperative complication was defined as an event associated with a clinically meaningful adverse visual outcome and/or requiring major additional intervention with potential visual consequences. The operating surgeon was consulted only when clarification of intraoperative context was required. The primary endpoint was the frequency and profile of intraoperative events with mechanistic interpretation and management, summarized in a standardized table (event type, micro-step, haptic involved, hypothesized mechanism, and corrective action). Secondary outcomes included operative timing-Yamane fixation time (from scleral marking to final intrascleral haptic burial) and total surgical time (from trocar insertion to trocar removal)-as well as postoperative safety and visual outcomes, including complications and corrected distance visual acuity (CDVA) at one week, one month, and last follow-up.

The structured checklist was developed as an exploratory educational self-audit tool and is intended to facilitate standardized documentation and future validation in broader cohorts. 

Statistical approach

Descriptive statistics were used to summarize event frequencies, procedural stages, mechanisms, and management. Continuous variables were summarized as mean ± standard deviation (SD) or median with interquartile range (IQR), as appropriate. Comparisons of continuous variables were performed using the independent-samples t-test or Mann-Whitney U test, as appropriate.

## Results

Baseline characteristics

A total of 24 eyes from 24 patients were included, representing the surgeon's early adoption of the Yamane technique. The mean age was 61.2 ± 12.1 years, and 16 patients (66.7%) were male. Aphakia and dislocated IOL were the most common surgical indications. Diabetes mellitus was present in five patients (20.8%). Pre-existing retinal comorbidities were present in seven eyes (29.2%), comprising diabetic retinopathy in two eyes (one moderate and one severe nonproliferative diabetic retinopathy), lattice degeneration in three eyes, a horseshoe retinal tear in one eye, and traumatic subretinal hemorrhage in one eye. Baseline demographic and ocular characteristics are summarized in Table 1.

**Table 1:** Baseline demographic and ocular characteristics of the study cohort. CDVA: corrected distance visual acuity; IOL: intraocular lens; logMAR: logarithm of the minimum angle of resolution; OD: right eye; OS: left eye; SD: standard deviation.

Characteristic	Value
Patients, n	24
Eyes, n	24
Age, years, mean ± SD	61.2 ± 12.1
Sex, n (%)	
Male	16 (66.7)
Female	8 (33.3)
Laterality, n (%)	
Right eye (OD)	16 (66.7)
Left eye (OS)	8 (33.3)
Surgical indication, n (%)	
Aphakia	6 (25.0)
Dislocated IOL	6 (25.0)
Dislocated lens	5 (20.8)
Subluxated lens	4 (16.7)
Subluxated IOL	2 (8.3)
Posterior capsule rupture	1 (4.2)
Axial length, mm, mean ± SD	23.99 ± 1.18
Preoperative CDVA, logMAR, mean ± SD	0.96 ± 0.79
Diabetes mellitus, n (%)	5 (20.8)

All procedures were completed successfully using the flanged intrascleral fixation. General anesthesia was used in 20 eyes (83.3%), while 4 eyes (16.7%) underwent surgery under peribulbar anesthesia. Posterior vitreous detachment (PVD) was present in 23 eyes (95.8%), comprising 10 eyes (41.7%) with pre-existing PVD and 13 eyes (54.2%) with intraoperatively induced PVD.

Intraoperative events

Intraoperative events occurred in 8 of 24 eyes (33.3%), predominantly related to haptic manipulation (Table 2). Four events occurred in the first 10 cases (40.0%) and four in the subsequent 14 cases (28.6%). Events in the first 10 cases were haptic-related, consisting of haptic slippage and haptic fracture, whereas later events included two cases of leading-haptic slippage, one case of vitreous hemorrhage, and one case of suprachoroidal hemorrhage. Overall, the most frequent event was haptic slippage (5 eyes, 20.8%), followed by haptic fracture (1 eye, 4.2%), mild vitreous hemorrhage (1 eye, 4.2%; self-limited), and suprachoroidal hemorrhage (1 eye, 4.2%; required postoperative drainage). All haptic-related events (6 eyes, 25.0%) were recognized and managed intraoperatively through haptic repositioning, re-threading, or scleral re-tunneling. Intraocular lens exchange was required in 1 eye (4.2%) due to haptic fracture. 

**Table 2:** Intraoperative events (n = 24 eyes). IOL: intraocular lens.

Event	n (%)	Stage of occurrence	Haptic involved	Hypothesized mechanism	Management
Haptic slippage	5 (20.8)	During haptic manipulation or externalization	Leading (4), trailing (1)	Insufficient threading length; optic rotation and torque during trailing-haptic docking with nondominant hand	Haptic reposition, re-threading, and scleral re-tunneling
Haptic fracture	1 (4.2)	During externalization	Leading	Excessive traction on trailing haptic causing stress on leading haptic	IOL exchange
Vitreous hemorrhage	1 (4.2)	During scleral tunneling	-	Possible pars plicata/ciliary process bleeding from a relatively anterior scleral tunnel.	Self-limited; no intervention
Suprachoroidal hemorrhage	1 (4.2)	After final haptic manipulation	-	Possible posterior ciliary-choroidal vessel injury during needle passage or haptic manipulation	Conservative approach intraoperatively. Surgical choroidal drainage two weeks post-op
Total	8 (33.3)		

Surgical duration

The mean Yamane fixation time was 17.0 ± 9.2 minutes (median 14.5; range 7-52) and decreased after the first ten cases (18.9 ± 13.3 minutes vs 15.6 ± 4.8 minutes, p > 0.05). Total surgical time, including vitrectomy and associated procedures, averaged 77.7 ± 21.6 minutes (median 77; range 38-125).

Postoperative outcomes

At a median follow-up of 12 months (range, 1-19 months), CDVA improved significantly from 0.96 ± 0.79 logMAR preoperatively to 0.47 ± 0.55 logMAR postoperatively (p < 0.05). Postoperative complications included transient optic capture (1 eye, 4.2%), which resolved spontaneously, and mild intraocular lens tilt (1 eye, 4.2%) with maintained corrected distance visual acuity of 20/20. The suprachoroidal hemorrhage occurred in 1 eye (4.2%), required surgical drainage, and resulted in limited final visual acuity (20/400). No cases of retinal detachment, glaucoma, endophthalmitis, or bullous keratopathy were observed. Macular optical coherence tomography (OCT) was available in 20 eyes. Among these, macular abnormalities were detected in 5 eyes (25.0%), including cystoid macular edema in 3 eyes (15.0%) and mild epiretinal membrane in 2 eyes (10.0%).

## Discussion

This retrospective series analyzed 24 cases during the early adoption of Yamane flanged intrascleral IOL fixation by a single vitreoretinal surgeon, focusing on intraoperative events and their mechanisms rather than final visual outcomes. Despite the technical demands inherent to early experience, all cases were successfully completed. The safety profile was comparable with previous studies [2,4,5,8-10].

**Figure 1:**
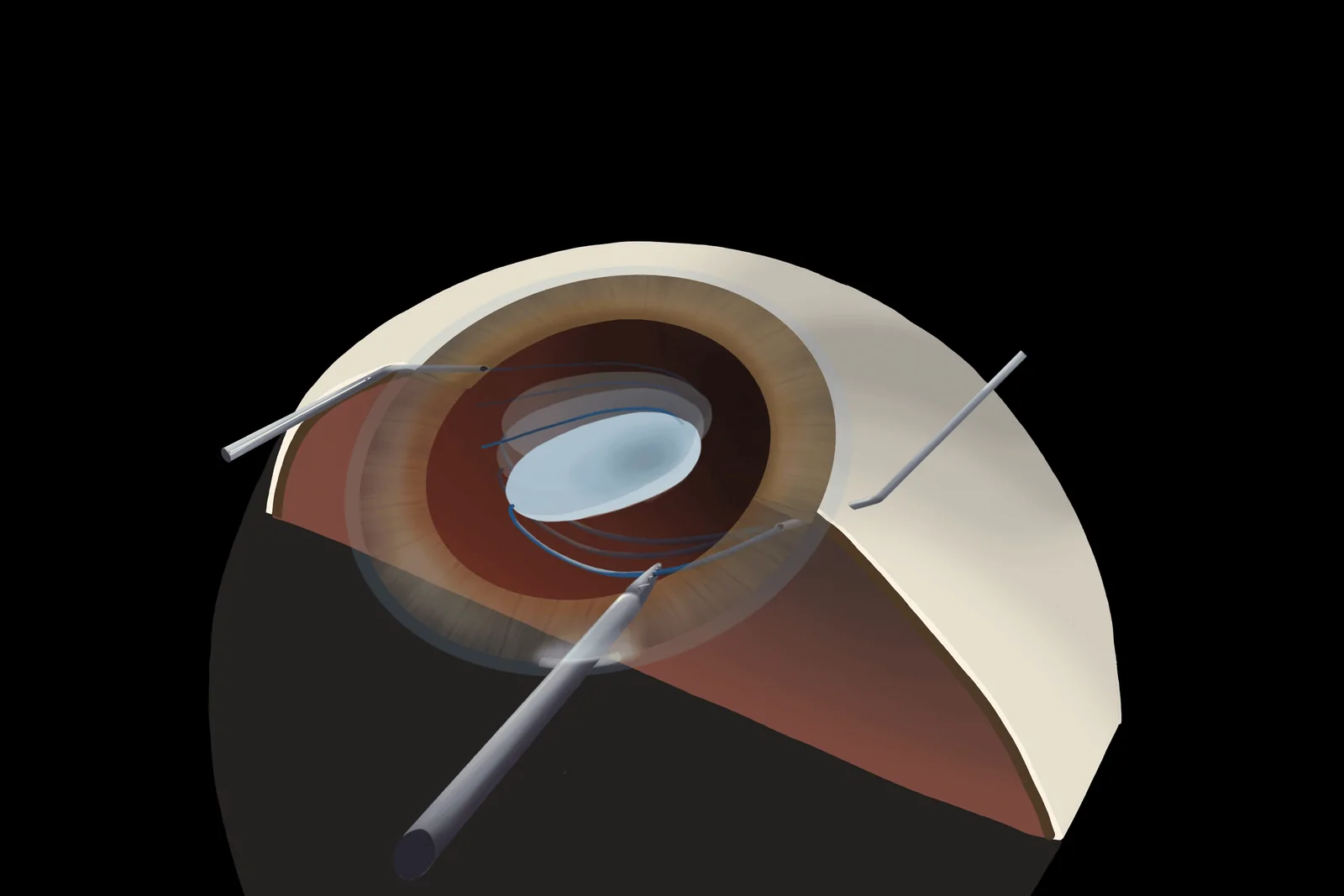
Hypothesized mechanism of leading-haptic slippage. During the “let-go” phase, temporary release of the first needle can allow nasal and downward needle rotation, altering the needle-leading-haptic alignment. Subsequent nondominant-hand trailing-haptic manipulation transmits optic torque to the IOL-haptic complex; together, these factors can result in disengagement of the leading haptic from the needle lumen. This figure is an original creation by the authors using the Sketchbook application for Android (Sketchbook, Inc., San Rafael, CA, USA).

Intraoperative events occurred in one-third of cases, consistent with Cheng et al. [4], who reported similar frequencies (33%) during early experience with sutureless fixation. However, in contrast to Cheng's report, most haptic-related events in this study were managed intraoperatively through haptic repositioning, re-threading, or scleral re-tunneling; IOL exchange was required in one case of haptic fracture. Video review identified a reproducible point of vulnerability for leading-haptic slippage immediately after leading-haptic threading, when the first needle is temporarily released during the “let-go needle” phase (Figure 1). At this moment, the needle tended to rotate nasally and drift downward once released. This shift in the needle-haptic axis increases the likelihood that the previously docked leading haptic will disengage from the needle lumen. Slippage most often occurred during subsequent trailing-haptic docking, which typically requires nondominant-hand manipulation at a steeper and less ergonomic working angle. When forceps-haptic-needle alignment is suboptimal, compensatory maneuvers may introduce whole IOL-needle complex rotation; with insufficient leading-haptic insertion depth, even small torsional forces transmitted through the optic-haptic complex can dislodge the leading haptic [11]. This risk may be further accentuated by the use of a 27-gauge needle rather than the 30-gauge thin-walled needle originally described, potentially reducing grip stability and alignment precision. Overall, these observations are consistent with prior reports that trailing-haptic control is a common technical challenge and help explain why multiple modifications have been proposed to improve stability during docking [11-14].

**Figure 2:**
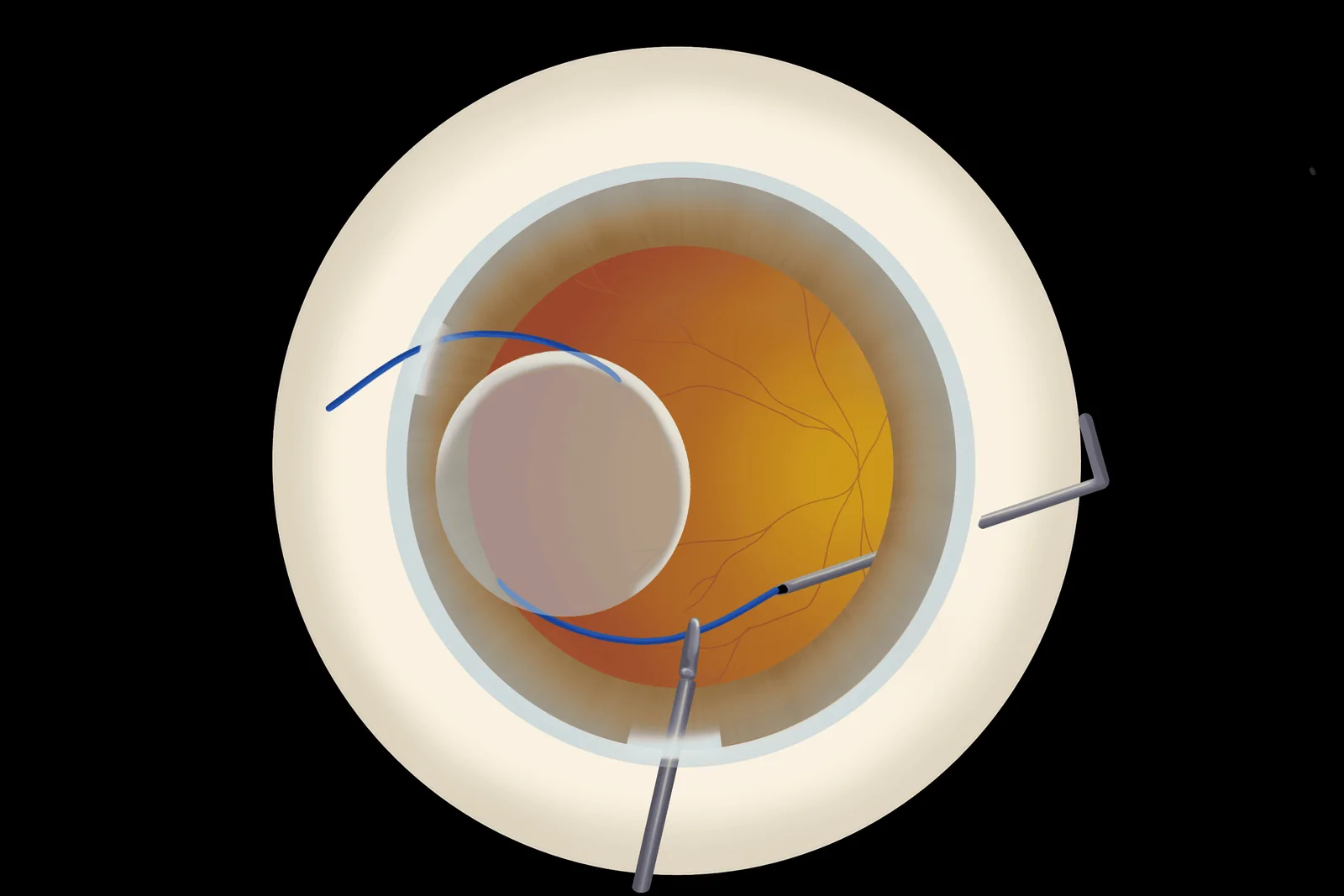
Kim's modification trailing-haptic-first to improve stability. Schematic illustration showing transcorneal externalization of the leading haptic for improving stability, followed by trailing-haptic threading and externalization. This figure is an original creation by the authors using the Sketchbook application for Android (Sketchbook, Inc., San Rafael, CA, USA); the concept was adapted from the technique description by Kim [11] and has not been reproduced or adapted from any previously published source.

Based on the structured video review, specific, teachable refinements were identified to mitigate these vulnerability points. The review emphasized ensuring that both leading and trailing haptics are threaded into the needle lumen (approximately half of the haptic length) and applying a trailing-haptic-first workflow, as described in the literature [11]. In this sequence, the leading haptic is temporarily externalized through the corneal incision to stabilize the optic during trailing-haptic docking, which was intended to limit optic rotation and reduce the risk of tilt or posterior drop (Figure 2). The trailing haptic is then threaded and externalized through the scleral tunnel, after which the leading haptic is reintroduced into the anterior chamber and docked into the needle lumen using the dominant hand.

Other than haptic-related events, we observed two hemorrhagic complications in our early adoption: one mild vitreous hemorrhage during scleral tunneling and one suprachoroidal hemorrhage after the final haptic manipulation. These events may relate to scleral tunnel positioning and needle trajectory. The pars plicata contains highly vascular ciliary processes, and major bleeding may occur when the needle is placed too close to the posterior surgical limbus [15,16]. The suprachoroidal hemorrhage required choroidal drainage two weeks postoperatively and will be reported separately in a dedicated article.

A complete vitrectomy before IOL fixation also enhanced safety by preventing vitreous entrapment of dislodged haptics and reducing the risk of retinal traction or detachment. The absence of postoperative retinal complications in this series may reflect the consistent completion of vitrectomy and meticulous wound suturing, both of which help maintain chamber stability and prevent hypotony [17].

Video-based analysis proved instrumental for detecting these subtle technical relationships that might otherwise be overlooked during live surgery. By objectively mapping the sequence of intraoperative steps and correlating them with complications, the review process enabled pattern recognition and progressive self-correction [7]. This deliberate, evidence-based reflection transformed the procedure from a set of isolated maneuvers into a coordinated and reproducible sequence in which the stability of the leading haptic determines the success of the trailing one.

From an educational standpoint, the key contribution of this work is the “audit-and-refine” scenario enabled by structured video review: objective identification of where an event occurs (micro-step localization), why it occurs (mechanistic attribution), and what is done to recover the case (corrective action), using a standardized checklist [7]. This approach supports pattern recognition and progressive self-correction beyond subjective recall, and it provides a reproducible framework that can be incorporated into competency-based teaching and supervision for fellows and residents learning sutureless scleral fixation [7]. In this sense, the structured tool is intended not to generalize a single-surgeon's event rates, but to standardize the process of reviewing and anticipating vulnerability points during early adoption-forming a practical platform for future multi-surgeon validation and curriculum development.

Several complications commonly reported during early Yamane adoption, such as IOL tilt/malcentration and exposed or insufficiently buried flanges, were not observed in this series. These are often linked to asymmetric tunnel geometry, unequal haptic capture/tension, or inadequate flange burial/oversized flanges. Their absence may reflect careful symmetric tunnel placement and flange construction, although the sample size and follow-up may have limited detection of rare or late-onset events.

This study's limitations include its small sample size, single-surgeon design, and single-reviewer video assessment, which may limit the generalizability of the observed event patterns and the reproducibility of the video-based classification across different surgeons and reviewers. The lack of objective imaging to quantify IOL tilt or decentration is another limitation. Variability in ocular comorbidities may also have influenced visual acuity outcomes. Patient-specific anatomical factors that could influence intraoperative ergonomics, such as scleral rigidity, were not systematically documented in the surgical records and therefore could not be evaluated. Future multicenter studies involving multiple surgeons and independent video reviewers would help validate the observed event patterns and assess the reproducibility of this analytical approach. Nonetheless, the findings provide practical insights into how and why events may occur during early adoption.

## Conclusions

Structured video review with micro-step classification helped localize intraoperative vulnerability points and link events to plausible mechanisms and recovery actions during early Yamane adoption. While this single-surgeon series cannot generalize event rates, the proposed checklist offers a practical framework for mentor feedback and targeted technical refinement during training. Future validation in broader cohorts is warranted.

## Appendices

Structured surgical video review tool for Yamane flanged intrascleral intraocular lens (IOL) fixation

Purpose

This tool standardizes surgical video review to identify intraoperative events, localize complication-prone micro-steps, infer plausible mechanisms, and document corrective strategies during early adoption of Yamane flanged intrascleral IOL fixation.

*Micro-step Map* 

**Table 3:** Micro-step map (M1-M17) for structured surgical video review of Yamane flanged intrascleral IOL fixation. IOL: intraocular lens.

Micro-step	Definition
M1	IOP maintenance/globe stabilization: Establish and maintain stable intraocular pressure throughout fixation using pars plana infusion and/or an anterior chamber maintainer.
M2	Vitreous management: Perform anterior vitrectomy and/or pars plana vitrectomy (PPV) as indicated to clear vitreous from the anterior chamber/haptic path and reduce vitreoretinal traction.
M3	IOL insertion into the anterior chamber, leaving the trailing haptic outside to prevent posterior dislocation.
M4	Scleral tunneling/needle placement--first side (leading side) (paired angled scleral tunnel/sclerotomy at the planned fixation point).
M5	Leading-haptic docking (engagement of the leading haptic tip into the first needle lumen).
M6	Leading-haptic threading (advancement within the needle lumen to achieve stable capture).
M7	“Let-go” phase (detachment of the first needle from the syringe, followed by temporary release/placement of the needle while preparing the opposite side).
M8	Scleral tunneling/needle placement-opposite side (trailing side) (paired angled scleral tunnel/sclerotomy at the planned fixation point).
M9	Trailing-haptic docking (engagement of the trailing haptic tip into the second needle lumen).
M10	Trailing-haptic threading (advancement within the needle lumen to secure capture).
M11	Trailing-haptic externalization (externalization of the needle-haptic complex through sclera).
M12	Trailing-haptic flanging (cautery to create the trailing flange).
M13	Trailing flange burial/tucking (seat/bury the trailing flange within the scleral tunnel).
M14	Leading-haptic externalization (return to the first needle and externalize the leading haptic).
M15	Leading-haptic flanging (cautery to create the leading flange).
M16	Leading flange burial/tucking (seat/bury the leading flange within the scleral tunnel).
M17	Wound closure (corneal and scleral closure as indicated, including suturing of corneal incisions and sclerotomies).

Definitions

Intraoperative event: a deviation from the ideal workflow requiring an unplanned maneuver, repeated attempt, or corrective action, even if resolved intraoperatively.

Intraoperative complication: an event associated with clinically meaningful harm/high-risk threat to visual acuity and/or requiring major additional intervention 

Data Fields to Capture (For Each Event)

• Case ID/Video ID

• Micro-step (M1-M17)

• Haptic side (Leading vs Trailing) (if applicable)

• Event type (taxonomy code)

• Mechanism tags (choose ≥1)

• Corrective action (what was done intraoperatively)

• Timestamp (optional)

• Notes (brief context)

Event Taxonomy (Minimum Core Set)

**Table 4:** Intraoperative event taxonomy and operational definitions for structured surgical video review. IOL: intraocular lens.

Code	Definition
E1	Haptic slippage/disengagement (Leading/Trailing)
E4	Haptic breakage/fracture
E5	Intraoperative vitreous hemorrhage
E6	Intraoperative choroidal hemorrhage/suprachoroidal hemorrhage (SCH)
E7	Conversion to alternative fixation/strategy
E8	Flange inadequate at end-of-case (optional)
E9	End-of-case IOL tilt/malcentration noted
E10	End-of-case exposed flange or insufficient flange burial (risk of extrusion)

Mechanism Tags (Select ≥1 Per Event)

**Table 5:** Mechanism tags (Mec1-Mec13) and definitions used for structured surgical video review. IOL: intraocular lens.

Tag	Mechanism
Mec1	Inadequate threading length
Mec2	Forceps-haptic-needle axis misalignment
Mec3	Nondominant-hand ergonomics/limited exposure
Mec4	Optic torque/rotation transmitted to leading haptic
Mec5	Tunnel geometry/suboptimal needle trajectory
Mec6	Bleeding or poor visualization
Mec7	Asymmetric nasal–temporal tunnel geometry/misaligned sclerotomies (IOL tilt risk)
Mec8	Unequal leading vs trailing haptic docking/capture (IOL tilt risk)
Mec9	Unequal flange burial depth/tension imbalance (IOL tilt risk) (optional)
Mec10	Inadequate flange burial (too superficial tucking) (flange exposure risk)
Mec11	Oversized/irregular flange configuration (flange exposure risk)
Mec12	Short scleral tunnel/thin sclera/conjunctival friction (flange exposure risk) (optional)
Mec13	Other (specify)

Corrective Action Categories

**Table 6:** Corrective action codes (C1-C7) and definitions used in structured surgical video review. IOL: intraocular lens.

Code	Corrective action
C1	Redocking/re-threading
C2	Re-grasp/instrument exchange
C3	Re-tunneling/repeat needle pass
C4	IOL exchange
C5	Hemostasis maneuver/raise IOP/tamponade
C6	Convert strategy (specify)
C7	Observation (self-limited)

Event Logging Sheet (Per Case/Video)

Complete one line per event observed. Leave blank if not assessable.

**Table 7:** Raw intraoperative event log template for structured surgical video review.

Case_ID	Video_ID	Micro-step (M#)	Haptic (L/T)	Event code	Mechanism tag(s)	Corrective action	Timestamp	Notes
